# Hypotensive effect of captopril on deoxycorticosterone acetate-salt-induced hypertensive rat is associated with gut microbiota alteration

**DOI:** 10.1038/s41440-021-00796-x

**Published:** 2021-12-02

**Authors:** Haicui Wu, Theo Y. C. Lam, Tim-Fat Shum, Tsung-Yu Tsai, Jiachi Chiou

**Affiliations:** 1 Department of Applied Biology and Chemical Technology, The Hong Kong Polytechnic University, Hung Hom, Kowloon, Hong Kong, China; 2 Research Institute for Future Food, The Hong Kong Polytechnic University, Hung Hom, Kowloon, Hong Kong, China; 3 Department of Civil and Environmental Engineering, The Hong Kong Polytechnic University, Hung Hom, Kowloon, Hong Kong, China; 4 Department of Food Science, Fu Jen Catholic University, New Taipei City, 24205 Taiwan

**Keywords:** Captopril:, DOCA, Hypertension, Gut microbiota

## Abstract

The role of the gut microbiota in various metabolic diseases has been widely studied. This study aims to test the hypothesis that gut microbiota dysbiosis is associated with DOCA-salt-induced hypertension, while captopril, an antihypertensive drug, is able to rebalance the gut microbiota alterations caused by hypertension. Treatment with captopril resulted in an approximate 32 mmHg reduction in systolic blood pressure (162.57 vs. 194.61 mmHg) in DOCA-salt-induced hypertensive rats, although it was significantly higher than that in SHAM rats (136.10 mmHg). Moreover, the nitric oxide (NO) level was significantly increased (20.60 vs. 6.42 µM) while the angiotensin II (Ang II) content (42.40 vs. 59.47 pg/ml) was attenuated nonsignificantly by captopril treatment in comparison to those of DOCA-salt-induced hypertensive rats. The introduction of captopril significantly decreased the levels of tumor necrosis factor-α (TNF-ɑ) and interleukin-6 (IL-6). Hypertrophy and fibrosis in kidneys and hearts were also significantly attenuated by captopril. Furthermore, gut microbiota dysbiosis was observed in DOCA-salt-induced hypertensive rats. The abundances of several phyla and genera, including Proteobacteria, Cyanobacteria, *Escherichia-Shigella*, *Eubacterium nodatum* and *Ruminococcus*, were higher in DOCA-salt-induced hypertensive rats than in SHAM rats, while these changes were reversed by captopril treatment. Of particular interest, the genera *Bifidobacterium* and *Akkermansia*, reported as beneficial bacteria in the gut, were abundant in only hypertensive rats treated with captopril. These results provide evidence that captopril has the potential to rebalance the dysbiotic gut microbiota of DOCA-salt-induced hypertensive rats, suggesting that the alteration of the gut flora by captopril may contribute to the hypotensive effect of this drug.

## Abstract (web summary)

Captopril was shown to improve RAAS, oxidative stress, and inflammation, and also rebalance dysbiotic gut microbiome, firstly elucidating its hypotensive effect via reverting gut microbiome.

## Graphical abstract

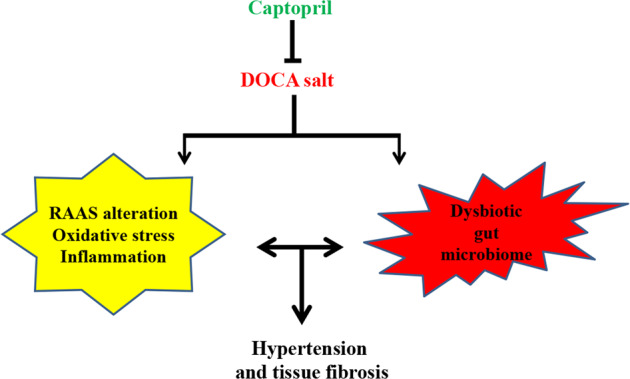

## Introduction

The gut microbiota, as an “essential organ” in humans, harbors approximately 150 times more genes than those found in the entire human genome [1, 2]. Increasing evidence indicates that the gut microbiota within humans plays crucial roles in human health and diseases, including obesity [3], colorectal cancer [4], liver cirrhosis [5], arthritis [6], and type 2 diabetes [7]. The gut microbiota is associated with energy harvesting by extracting energy from food and could promote obesity [8]. Many metabolic genes found in the gut microbiota encode unique and specific enzymes that are involved in different biochemical pathways in humans [9]. Moreover, the gut microbiota protects its host from infection by foreign pathogens via various strategies, such as providing physical barriers and immunomodulation [10]. This evidence demonstrates that microbial symbiosis in the human gut has a close relationship with different diseases.

Hypertension, one of the most prevalent global public health concerns, is a risk factor for heart disease, stroke, and death [11]. Chronic kidney disease [12], age [13], overweight [14], and genetics [15, 16] are potential factors that cause hypertension. Various approaches, including altering dietary habits [17], regular physical exercise [18], reducing stress [19], and medication use, have been suggested to treat hypertension. Drugs, such as diuretics [20], calcium-channel blockers [21], peripheral adrenergic inhibitors [22], and angiotensin-converting enzyme (ACE) inhibitors [23], are commonly used. Among all the factors contributing to regulating blood pressure, the renin-angiotensin-aldosterone system (RAAS) is regarded as one of the most important pathways [16]. Captopril, a commercial antihypertensive drug, is an ACE inhibitor that blocks the conversion of Ang I into Ang II [24–26]. It has been suggested that captopril provides sustained blood pressure control with minimal side effects during long-term therapy for hypertension [27].

Elevated blood pressure observed in germ-free mice receiving fecal microbiota transplantation (FMT) from hypertensive patients revealed a link between the gut microbiome and hypertension [28]. Moreover, the Firmicutes-to-Bacteroidetes ratio was recently reported to be increased in spontaneously hypertensive rats (SHRs) [28], Ang II infusion rats [29], and a small group of humans with essential hypertension [28]. Acetate- and butyrate-producing bacteria have been shown to be depleted in SHRs, while an overgrowth of bacteria such as *Prevotella* and *Klebsiella* has been found in both prehypertensive and hypertensive populations [28].

Another widely studied hypertensive animal model is the DOCA-salt-induced hypertensive rat, which is one of the neurogenic models of hypertension illustrating the crosstalk between the nervous system and hormones and is a kidney model due to an imbalance of renal sodium handling in which greater amounts of sodium and water are reabsorbed by the kidney, resulting in hypervolemia [30]. It has been reported that gut microbiota dysbiosis was linked to DOCA-salt-induced hypertension in mice, while a high-fiber diet and acetate effectively reduced blood pressure by rebalancing the gut microbiota and increasing the prevalence of *Bacteroides acidifaciens* [31]. Another end-product produced by intestinal bacteria, β-hydroxybutyrate, has also been found to be capable of ameliorating hypertension in high-salt diet-fed hypertensive rats [32].

This study aims to elucidate the blood pressure-lowering effect of captopril on DOCA-salt-induced hypertensive rats from the view of the gut microbiota and determine the role of gut microbiota dysbiosis in hypertension. We hypothesize that the hypotensive effect of captopril on DOCA-salt-induced hypertension is partially attributed to improving the dysbiotic gut microbiota in addition to its effect on the host physiological response [33, 34].

## Methods

### Animals

The experimental procedures used in this study were approved by the Animal Subject Ethics Committee of The Hong Kong Polytechnic University and were conducted in accordance with Hong Kong Government Animal Ordinance Chapter 340. A total of 15 male Sprague–Dawley (SD) rats weighing 180–200 g (~6 weeks old) were purchased from Centralized Animal Facilities (CAF) of Hong Kong Polytechnic University and housed individually in a temperature-controlled room (at a temperature of 21 ± 2 °C and 55% ± 10% relative humidity) with a 12:12 h light-dark cycle. They were supplied with autoclaved corn-cob bedding (Lab Supply) and ad libitum irradiated diets (LabDiet 5053). The rats were housed for a minimum of two weeks in the facility prior to being used in this experiment, and there were 5 rats in each group.

### DOCA-salt-induced hypertensive rat model

Three groups, namely, SHAM (normotensive rats), DOCA (DOCA-salt-induced hypertensive rats without treatment), and CAP (DOCA-salt-induced hypertensive rats with captopril administration), were used in this study. Hypertension was induced for 14 weeks by subcutaneous injection of DOCA (Sigma, 20 mg/kg, St. Louis, MO, USA) twice per week, in combination with 1.0% NaCl and 0.2% KCl in the drinking water in both the DOCA and CAP groups. Rats in the SHAM group were injected with saline, and tap water was supplied. In the CAP group, hypertensive rats induced by DOCA-salt were given captopril (Sigma, 50 mg/kg, St. Louis, MO, USA) by oral gavage daily for the last 5 weeks. Fecal samples were collected at the end of the experiment.

### Measurement of body mass, water intake, food intake, and blood pressure

The body mass and intake of food and water were measured weekly. Blood pressure was recorded biweekly by the tail-cuff method using a BP-2000 Blood Pressure Analysis System (Visitech System, Inc., Apex, NC, USA). Rats were kept at 37 °C for 10 min to detect pulsations in the tail artery prior to measurement. Ten readings were taken, and the mean of all measurements was calculated. To minimize stress-induced variations in blood pressure, the same person performed all the measurements in the same environment at the same time during the days. A training period of 2 weeks was established before the actual trial time to accustom the rats to the procedure.

### Cardiac ultrasound

Rats were anesthetized using 3% isoflurane, and echocardiography was performed using a FUJIFILM VisualSonics Vevo LAZR Multimodality Imaging Platform with a Transducer LZ250 transducer. The left ventricle internal diameter was determined.

### Analysis of biochemical parameters

All rats were sacrificed by CO_2_ inhalation after fasting for 24 h. Blood was collected by cardiac puncture. Plasma was obtained by collecting blood in heparinized syringes containing 5% heparin and stored at −80 °C. The aorta, heart, brain, and kidney tissues were harvested, weighed, and stored at −80 °C. NO (Cayman Chemical Company, Ann Arbor, USA) and Ang II (Sigma–Aldrich Co., St. Louis, MD, USA) in plasma and IL-6 and TNF-ɑ in the left half of the brain (Biolegend, Inc., San Diego, CA) were detected by ELISA kits.

### Histology

Tissues were fixed in 10% neutral buffered formalin, embedded in paraffin, and sectioned to 5 µm thickness. The heart and kidney were stained with a Trichrome Stain (Masson) Kit (Sigma, St. Louis, MO, USA) to determine collagen fibrosis. The aorta was stained with an Elastic Stain Kit, a modified Verhoeff Van Gieson Elastic Stain Kit (Sigma, St. Louis, MO, USA). Quantification was performed in 3 random fields of view per section with an optical microscope (×400 magnification). Renal and cardiac perivascular fibrosis was analyzed/graded from 0 to 4 by using a histology damage score (0, no lesion; 1, focal lesion; 2, multifocal mild lesion; 3, multifocal moderate lesion; and 4, diffuse, moderate, or severe damage) [35]. The percentage of tubulointerstitial damage and vascular elastin distribution in the aorta were quantified by ImageJ software [36, 37].

### Analysis of the gut microbiota

Stool samples were collected sterilely from the rectum at week 14 and stored at −80 °C. DNA was extracted from each sample using a TIANamp Stool DNA Kit (Tiangen, Beijing, China), and the V3-V4 region of the bacterial 16 S rRNA gene was sequenced by an Illumina HiSeq (300–500 bp paired-end reads) at Majorbio BioPharm Tech. Co. (Shanghai, China). Raw data were filtered, and primers were trimmed. The results of the sequencing were analyzed using QIIME 2, and figures were generated using the i-sanger platform. PICRUSt 2 was used to predict the molecular functions of the microbiota from the known sequence databases KEGG and MetaCyc [38].

### Short-chain fatty acid (SCFA) content in feces

The concentration of fecal SCFAs was determined by gas chromatography (GC). A total of 0.15 g of feces was homogenized with 0.6 mL of Milli-Q water and centrifuged at 12,000 rpm for 10 min. The supernatants were filtered through a 0.22 µm nylon filter and acidified to pH 2.0–3.0 by adding HCl. SCFA levels in supernatants were measured by a GC Agilent 7890 (Santa Clara, CA, USA). The amount of major SCFAs in the feces was determined using the equation based on the standard curves derived from serially diluted known concentrations of acetate, propionate, butyrate, and isobutyrate.

### Fecal microbiota transplantation

Cecal and colon contents were collected and pooled from rats in the DOCA and SHAM groups after CO_2_ euthanasia. Each of the samples was diluted 1:5 in sterile PBS and centrifuged at 1000 rpm for 5 min, and the supernatant from each sample was aliquoted and frozen at −80 °C. The recipient received an oral gavage with 1 ml of a broad-spectrum antibiotic cocktail consisting of ampicillin, gentamycin, metronidazole, neomycin (each at 0.25 mg/ml), and vancomycin (0.125 mg/ml) once daily for 10 consecutive days. Two days after the last antibiotic administration, 1 ml of the pooled cecal/colonic supernatant was given to the recipient rats daily (SHAM → SHAM and DOCA → SHAM) for 4 consecutive days and weekly thereafter. The rats in the other two groups, SHAM and DOCA, were administered sterile PBS. This FMT treatment continued for 4 weeks.

### Data analysis

All values represent the means and standard errors of four-to-six independent experiments. Data were then compared using one-way ANOVA with Duncan’s multiple range analysis in SPSS statistical analysis software (IBM Software, Armonk, NY, USA).

## Results

### Protective effects of captopril on blood pressure and biochemical parameters in DOCA-salt-induced hypertensive rats

**Fig. 1:**
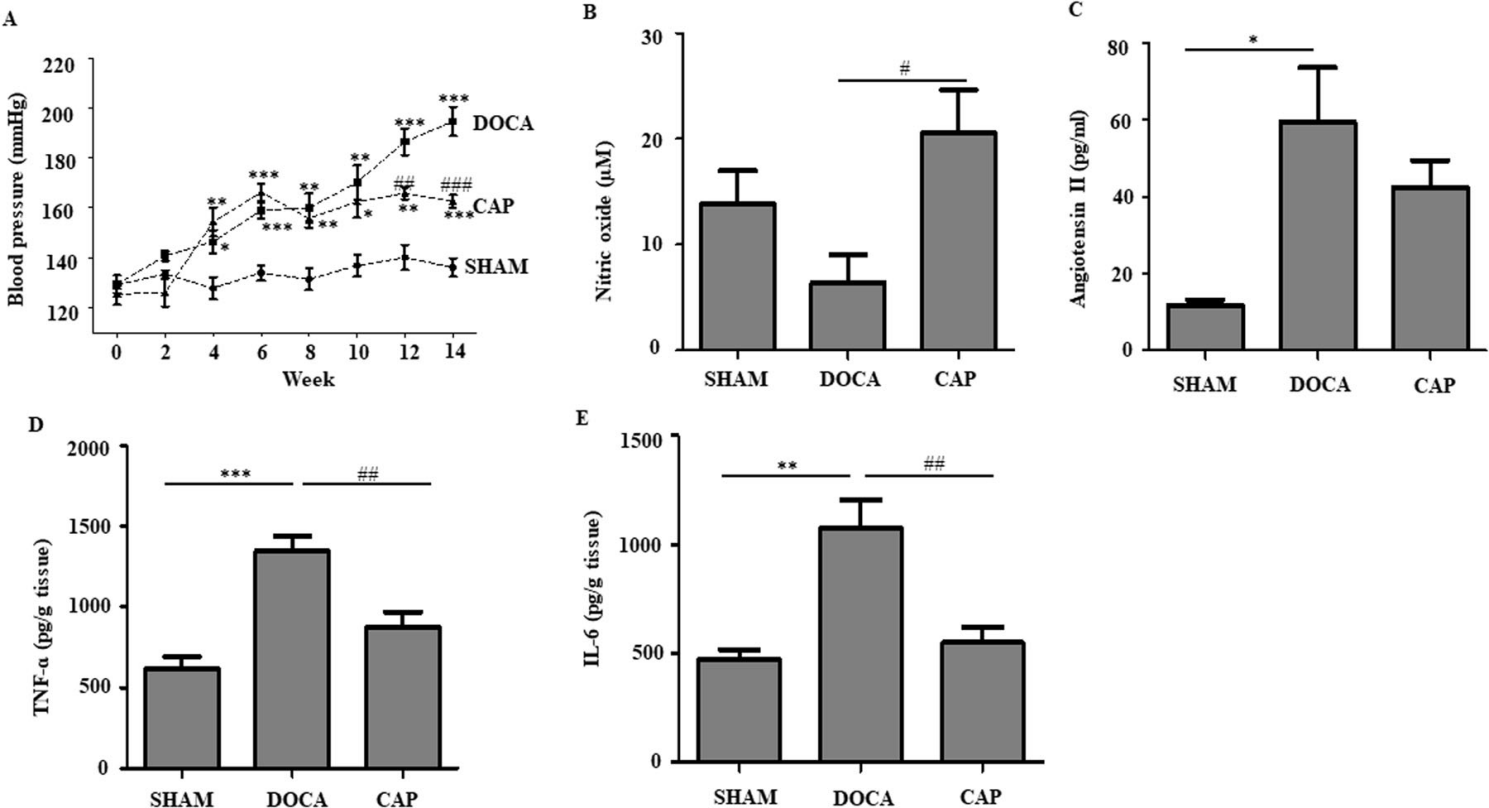
Effect of captopril on blood pressure and biochemical parameters in DOCA-salt-induced hypertensive rats. **A** Blood pressure. **B** NO content. **C** AngII level **D** TNF-ɑ. **E** IL-6 concentrations. *p < 0.05, **p < 0.01 and ***p < 0.001 vs. SHAM; ^#^*p* < 0.05, ^##^*p* < 0.01 and ^###^*p* < 0.001 vs. DOCA; *n* = 5

Captopril, an inhibitor of ACE, is an effective commercial drug for reducing blood pressure in hypertensive patients. To evaluate the effect of captopril on our DOCA-salt-induced hypertensive rat model, the rats were given captopril at week 9, when all the rats were successfully induced to develop hypertension. The water intake of rats in the DOCA and CAP groups was much higher than that in the SHAM group due to salt supplementation in the drinking water. However, rats in the CAP group had relatively less water intake than those in the DOCA group (Supplementary Figure 1A). No trends or differences were found for food intake in any of the three groups (Supplementary Figure 1B). The rate of body mass increase in the DOCA and CAP groups declined in comparison to that in the SHAM group (Supplementary Figure 1C). Blood pressure, the most important parameter evaluated, was significantly higher in the DOCA and CAP groups than in the SHAM group. As expected, the hypotensive effect was significant at week 12 in rats treated with captopril in comparison to those in the DOCA group (162.57 vs. 194.60 mmHg; Fig. 1A). Overall, captopril showed hypotensive action in the DOCA-salt-induced hypertensive rat model.

Impaired NO bioavailability is implicated in arterial stiffness, a major mechanism of systolic hypertension. Captopril administration in hypertensive rats significantly increased NO levels compared to those in DOCA-salt-induced hypertensive rats (Fig. 1B). A significantly higher Ang II level was observed in the DOCA group than in the SHAM group. The Ang II content decreased nonsignificantly after captopril administration in comparison to that in the DOCA group (Fig. 1C). Metabolic diseases, including hypertension, are closely linked to chronic inflammation. Hence, we also monitored the levels of some inflammatory factors in this study. The levels of the proinflammatory cytokines TNF-ɑ and IL-6 increased significantly in DOCA-salt-induced hypertensive rats compared to those in the SHAM rats, while captopril administration attenuated their levels (Figs. 1D and 1E). Our data demonstrated that captopril was able to improve certain parameters involved in the RAAS and inflammatory stress in the DOCA-salt-induced hypertensive rat model.

### Protective effects of captopril administration on the kidney and heart in DOCA-salt-induced hypertensive rats

**Fig. 2:**
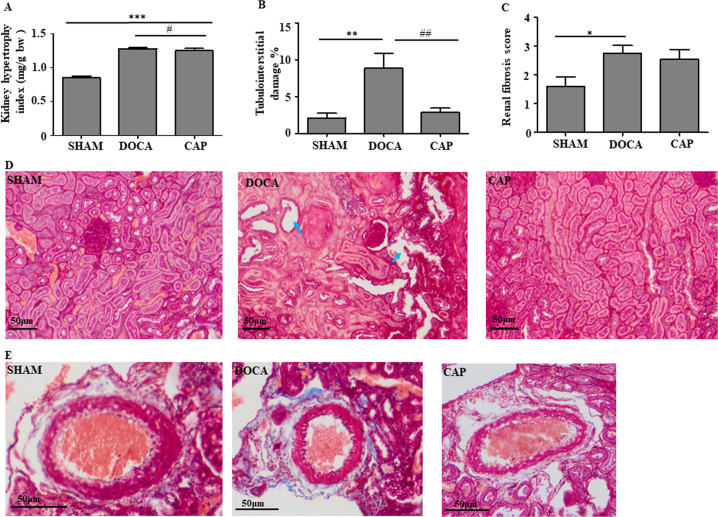
Effect of captopril on kidney changes in DOCA-salt-induced hypertensive rats. **A** Kidney hypertrophy index. **B** Quantitative percentage of tubulointerstitial damage. **C** Quantitative level of renal fibrosis. **D** Representative histological images of tubulointerstitial damage. **E** Perivascular fibrosis. The results were compared by one-way ANOVA and Duncan’s post hoc test; *p < 0.05, **p < 0.01 and ***p < 0.001. *ns* Not significant; *n* = 5

Kidney and heart hypertrophy are two features in the DOCA-salt-induced hypertensive model. By comparing the kidney index (the ratio of kidney weight to body mass), the values for the DOCA and CAP groups significantly increased compared to that for the SHAM group (Fig. 2A). Significant improvement in kidney hypertrophy was observed after the administration of captopril (Fig. 2A). Histological analysis showed that the percentage of tubulointerstitial damage of kidneys in the DOCA group was much higher than that in the SHAM group, and the damage was significantly reversed by captopril treatment (Fig. 2B,D). Furthermore, the renal perivascular fibrosis level was higher in the DOCA group than in the SHAM group, whereas the level was relatively but nonsignificantly attenuated after captopril administration, as noticed in the CAP group (Figs. 2C, E).

**Fig. 3:**
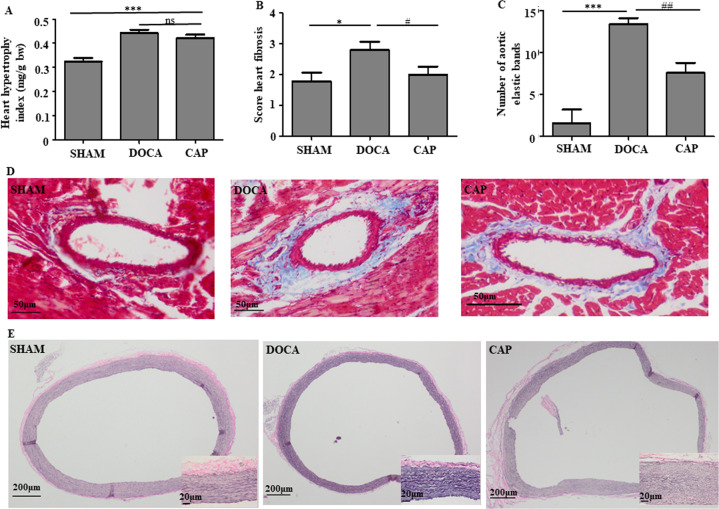
Effect of captopril on heart changes in DOCA-salt-induced hypertensive rats. **A** Heart hypertrophy index. **B** Quantitative level of heart fibrosis. **C** Quantitative level of aortic elastic bands. **D** Representative histological images of heart perivascular fibrosis. **E** Aortic fibrosis. The results were compared by one-way ANOVA and Duncan’s post hoc test; *p < 0.05, **p < 0.01, and ***p < 0.001. *ns* Not significant; *n* = 5

Similarly, the heart hypertrophy index of the DOCA group was significantly higher than that of SHAM group, while it was only slightly but nonsignificantly improved in the CAP group (Fig. 3A). Consistent with the changes observed in heart morphology, the echocardiographic evaluation showed that the internal dimension of the diastolic left ventricle (LVIDd) in the DOCA group was significantly increased compared with that in the SHAM group, while the intake of captopril in DOCA-salt-induced hypertensive rats notably ameliorated LVIDd dilation (Table 1). No significant differences were found for the internal dimension of the systolic left ventricle (LVIDs) in the three groups, although the LVIDs value was slightly reduced after the administration of captopril (Table 1). Furthermore, a comparison of the fibrosis levels in the heart vasculature showed that captopril significantly attenuated cardiac perivascular fibrosis (Figs. 3B and 3D). Similar results were further found in the aorta, in which the SHAM rats exhibited less elastic fibrosis than those in the DOCA group, and aortic fibrosis was improved after captopril administration (Figs. 3C and 3E). These results suggested that captopril improved the hypertrophy and fibrosis levels of various tissues in DOCA-salt-induced hypertensive rats.

**Table 1:** Effects of captopril administration on the thickness of the left ventricle in DOCA-salt-induced hypertensive rats

	SHAM	DOCA	CAP
LVIDd (mm)	6.54 ± 0.37	7.61 ± 0.03*	6.89 ± 0.29^#^
LVIDs (mm)	2.40 ± 0.31	2.73 ± 0.27	2.31 ± 0.45

The results were compared by one-way ANOVA and Duncan’s post hoc test; *p < 0.05 vs. SHAM; ^#^*p* < 0.05 vs. DOCA; *n* = 5

### Effect of captopril on the gut microbiota of DOCA-salt-induced hypertensive rats

**Fig. 4:**
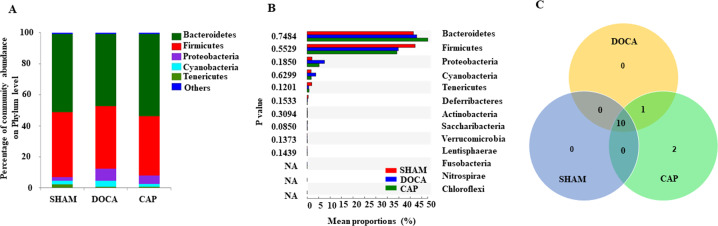
Differences in the gut microbiota at the phylum level in the SHAM, DOCA, and CAP groups at week 14. **A** Unique phyla of different groups. **B** Community abundance at the phylum level in different groups. One-way ANOVA bar plot of communities at the phylum level (**C**) in different groups

Alterations in the gut microbiota in all three groups were determined using 16 S rRNA sequencing. In this study, the alpha-diversity and richness indices among different groups did not show significant differences (data not shown). The microbial compositions of the three groups were similar in terms of relative abundance at week 0, showing a consistent baseline of fecal samples to start (Supplementary Figure 2). After 14 weeks of the experiment, the most prominent bacterial phyla in the SHAM group exhibited noticeable changes, namely, the F/B ratio was reduced from 1.49% to 1.02%, whereas Firmicutes and Bacteroidetes together contributed to 97% and 94% of the gut microbiota at weeks 0 and 14, respectively. These observations suggested that aging played an important role in the effects on the gut microbiota; therefore, the proper control for the gut microbiota should be that of SHAM rats at week 14 instead of the gut microbiota results obtained from the rats at week 0 (Fig. 4B and Supplementary Figure 2B). By comparing the relative abundances of the top five most abundant phyla in those three groups at week 14, no significant differences were found in Bacteroidetes and Firmicutes abundances (Fig. 4A). Proteobacteria and Cyanobacteria showed relatively higher abundances in the DOCA group than in the SHAM group, yet their abundances decreased after captopril treatment, although the changes were not significant (Fig. 4A, B). One bacterial phylum found in both the DOCA and CAP groups but not in the SHAM group was Fusobacteria, in which several species have been reported to cause human diseases, including periodontal diseases, Lemierre’s syndrome, and topical skin ulcers [39] (Fig. 4C). Two unique phyla observed only in the CAP group were Nitrospirae and Chloroflexi (Fig. 4C).

**Fig. 5:**
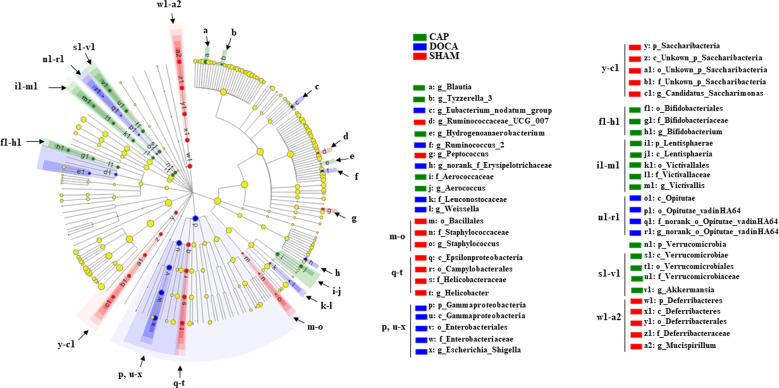
Phylogenetic distribution of the gut microbiota in the SHAM, DOCA, and CAP groups at week 14. Cladogram showing the phylogenetic distribution of the bacterial lineages in different groups. Circles indicate phylogenetic levels from phylum to genus. The diameter of each circle is proportional to the abundance of the group. Different-colored regions represent different constituents

A cladogram provided direct visualization to specify the altered communities at the phylum and genus levels in the gut microbiota (Fig. 5). All changes in the abundances of the bacteria illustrated in this figure were statistically significant. *Staphylococcus* (from order to genus), *Helicobacter* (from class to genus), *Candidatus Saccharimonas,* and *Mucispirillum* (from phylum to genus), and the genera *Ruminococcaceae UCG007* and *Peptococcus* were enriched in the SHAM rats. *Escherichia_Shigella* (from phylum to genus), norank genus belonging to the class Opitutae (from class to genus), and the genera *Eubacterium nodatum* group, *Ruminococcus_2* and norank belonging to the family Erysipelotrichaceae were abundant in the DOCA group. In the case of the CAP group, the abundances of *Bifidobacterium* (from order to genus), *Victivallis* (from phylum to genus), *Akkermansia* (from phylum to genus), *Aerococcus* (family and genus), and the genera *Blautia, Tyzzerella_3,* and *Hydrogenoanaerobacterium* were higher than those in the other two groups (Fig. 5). These results showed that the microbial communities in the gut of the three groups were dissimilar and enriched with different bacteria.

The most abundant bacteria in the CAP group were the genera *Blautia, Hydrogenoanaerobacterium,* and *Tyzzerella_3*, while *Escherichia_Shigella* (from phylum to genus) were the top enriched bacteria in the DOCA group. The order Campylobacterales and genus *Helicobacter* (from class to genus) were the most abundant bacteria in the SHAM rats (Supplementary Figure 3). Interestingly, the gut microbiota in the DOCA group was more linked to the phylum Gammaproteobacteria than to the other phyla, which includes many pathogenic strains. Nonetheless, the abundances of *Bifidobacterium* and *Akkermansia*, two genera reportedly beneficial for human health, were significantly higher in the CAP group than in the other 2 groups.

To further confirm that blood pressure is linked to the gut microbiota in our DOCA-salt-induced hypertension model, we conducted fecal microbiota transplantation from DOCA-salt-induced hypertensive rats and control rats to normotensive SD rats treated with a broad spectrum-antibiotic cocktail, namely, DOCA → SHAM and SHAM → SHAM. The blood pressure of DOCA → SHAM rats exhibited a significant increase in comparison with that of SHAM → SHAM rats (Supplementary Figure 4). This result indicated that the gut microbiota played an important role in blood pressure regulation in DOCA-salt-induced hypertension.

### The potential effect of captopril on gut microbiota-derived metabolites

**Fig. 6:**
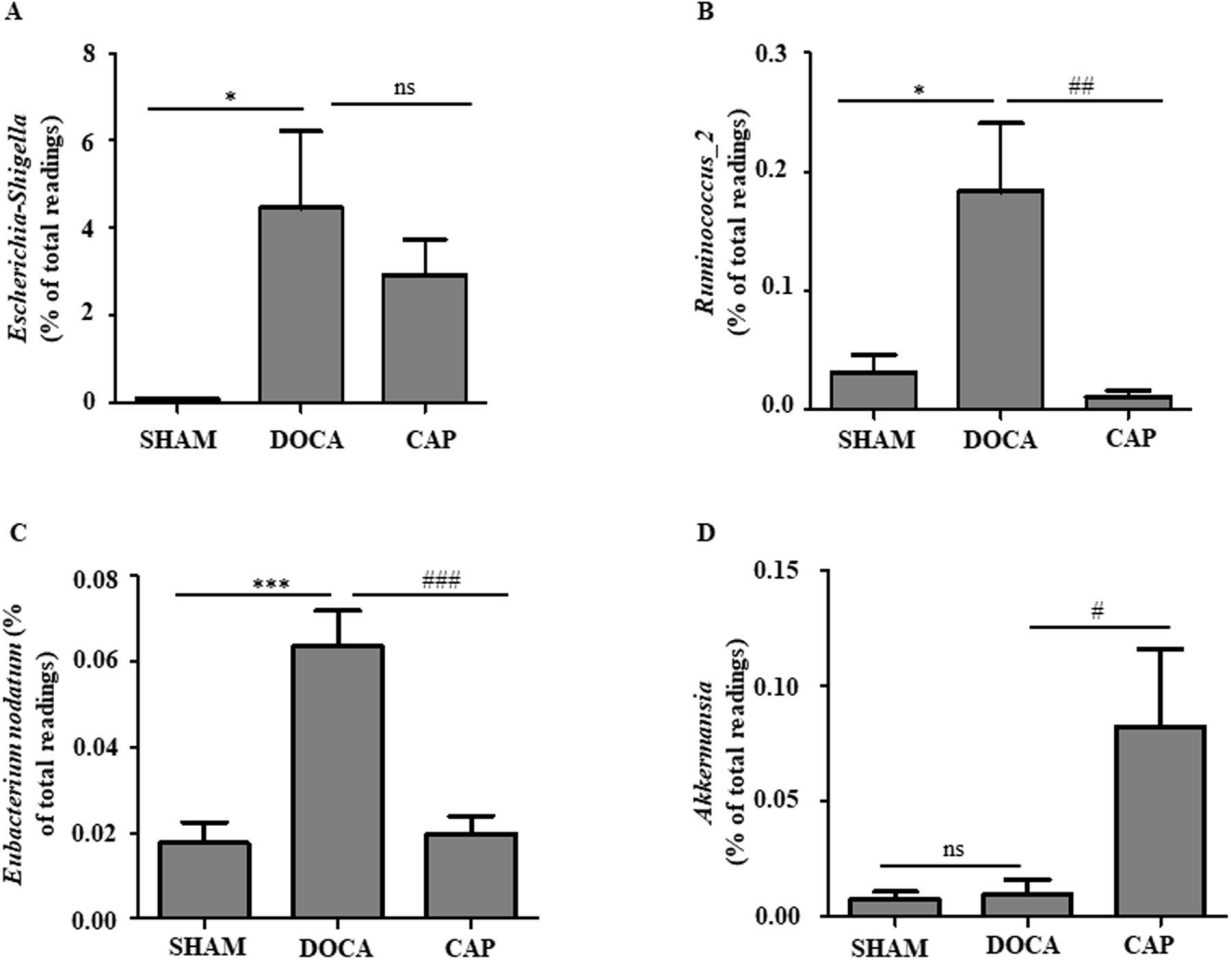
Effect of captopril on the abundance of several genera in the gut microbiota of DOCA-salt-induced hypertensive rats at week 14. **A** *Escherichia-Shigella*. **B** *Ruminococcus_2*. **C** *Eubacterium nodatum*. **D** *Akkermansia*. The results were compared by one-way ANOVA and Duncan’s post hoc test; *p < 0.05, **p < 0.01, and ***p < 0.001. *ns* Not significant; *n* = 4

Some genera of the gut microbiota were rebalanced by captopril treatment in DOCA-salt-induced hypertensive rats. *Escherichia-Shigella* were enriched in rats with hypertension (from 0.69% to 4.45%), yet their abundance was relatively attenuated to 2.90% in the CAP rats, although the change was nonsignificant (Fig. 6A). Similarly, captopril administration significantly restored the abundance of *Eubacterium nodatum* and *Ruminococcus_2*, two genera abundant in the DOCA group, from 0.063% and 0.183% to 0.019% and 0.010%, respectively (Figs. 6B and C). On the other hand, the abundance of *Akkermansia* was much higher in the CAP group than in the SHAM and DOCA groups (Fig. 6D). These bacteria might be associated with DOCA-salt-induced hypertension and the hypotensive effects of captopril.

Earlier studies have shown that SCFAs produced by the gut microbiota is closely associated with several physiological functions in humans [40]. It has been reported that acetate and butyrate fermenters were depleted while lactate-producing bacteria were enriched in the gut microbiota of SHRs [41]. Our data showed that the fecal contents of acetate, propionate, and butyrate were lower in the DOCA group than in the SHAM group and that their contents in the CAP group were reverted to levels similar to those in the SHAM group, although the changes were not statistically significant (Supplementary Figure 5A).

The gut microbiota participates in metabolizing certain amino acids to neurotransmitters, such as serotonin and gamma-aminobutyric acid (GABA), via tryptophan and glutamate metabolism, respectively, and both play major roles in host physiology and the pathophysiology of several diseases [42]. The abundance of bacteria associated with tryptophan degradation and biosynthesis, glutamate degradation, arginine degradation, and tyrosine degradation varied between the SHAM and DOCA groups, while captopril relatively but nonsignificantly restored their abundance (Supplementary Figure 5B-F). Overall, these results implied that the changes in the abundances of bacteria involved in the metabolism of certain amino acids might be associated with DOCA-salt-induced hypertension.

## Discussion

It has been shown that captopril-induced reduction in blood pressure was associated with the gut-brain axis, in which captopril was given to SHRs followed by monitoring the changes in the gut microbiota and neuronal activity [43]. However, the microbial composition of the gut flora was distinctly different among WKY rats and SHRs. Moreover, the aging factor was ignored during captopril treatment. Therefore, we aimed to study the effect of captopril in the DOCA-salt-induced hypertensive model, which has normalized background factors, including genetics and aging. To the best of our knowledge, this is the first report showing that the antihypertensive drug captopril rebalances the changes in the gut microbiota in a DOCA-salt-induced hypertensive rat model. The major findings in this study are as follows. (1) Captopril reduced blood pressure in DOCA-salt-induced hypertensive rats accompanied by increased Ang II, NO, and inflammatory responses and attenuated heart and kidney hypertrophy and fibrosis. (2) Two unique phyla found in the gut microbiota of the CAP group were Nitrospirae and Chloroflexi. (3) Captopril administration significantly reduced the abundances of *Eubacterium nodatum* and *Ruminococcus_2* and the abundance of *Escherichia-Shigella* was relatively reverted compared with those in the DOCA group. (4) *Bifidobacterium* and *Akkermansia* were more abundant in the CAP group than in the SHAM and DOCA groups. (5) The abundances of bacteria involved in the metabolism of several amino acids were partially reverted by captopril, although the changes were nonsignificant. These findings provide some indications that the hypotensive effects of captopril on hypertension were mediated not only through regulation of the RAAS and inflammation but also by rebalancing the gut microbiome to a certain extent.

Hypertension is a significant cause of morbidity and mortality worldwide. The most commonly studied hypertensive animal models include SHRs, salt-sensitive rats, Ang II infusion rats, and DOCA-salt-induced hypertensive mice and rats [29, 31]. Compared to hypertensive animal models generated by genetic manipulations and breeding selection, DOCA-salt-induced hypertension caused by an overreaction of the sympathetic nervous system and an imbalance in the RAAS is ideal to study essential hypertension [44]. The kidneys have the capacity to handle volume overload through water and salt excretion. Excess salt intake might impair renal function to induce hypertrophy in DOCA-salt-induced hypertensive rats. Angiotensin II (Ang II) acts on Ang II type 1 receptor (AT1) to induce various biological effects, including cytoskeleton remodeling, extracellular matrix generation, the inflammatory response, macrophage infiltration, fibroblast proliferation, tubule epithelial cell differentiation, and apoptosis in the kidney, all of which may interact with each other and eventually lead to hypertension and renal fibrosis [45]. Captopril, as an antihypertensive drug, might ameliorate renal damage by inhibiting the production of Ang II. DOCA is the precursor of aldosterone. Chronic infusion of aldosterone has been shown to increase the activity and mRNA level of calcineurin, a Ca2 + -dependent phosphatase, in the heart, contributing to cardiac hypertrophy [46]. Aldosterone also induces cardiovascular fibrosis. The mechanisms are still unclear but involve interactions among aldosterone, mineralocorticoid receptor, angiotensin II, AT1 receptor, ACE, and transforming growth factor-β1 [47]. ET-1 also plays a role in vascular growth [48] and in collagen deposition induced by mineralocorticoids such as DOCA [49] and by aldosterone [50] in cardiovascular tissues. The improvement of cardiac hypertrophy and fibrosis induced by captopril might be due to the ameliorated expression levels of components in the RAAS.

The role of the gut microbiome in various diseases, including obesity, colorectal cancer, liver cirrhosis, and type 2 diabetes, has drawn considerable attention [3–5, 7]. Nonetheless, in comparison to that in the aforementioned diseases, the relationship between the gut microbiota and hypertension, as well as the promising mechanisms behind this relationship, remain unclear. The direct shift of the gut microbiota in recipients toward the gut microbiota of fecal donors by FMT is regarded as a robust tool to reveal the causative role of the gut microbiota in diseases. Previous studies have shown that blood pressure was elevated in germ-free mice receiving FMT from hypertensive patients [28] and antibiotic-treated Wistar-Kyoto rats transplanted with the microbiota of stroke-prone SHRs (SHRSPs) [51]. In this study, we further confirmed that the blood pressure of the control rats increased from 132.37 to 150.82 mm Hg after receiving the fecal microbiota of DOCA-salt-induced hypertensive rats, providing evidence that DOCA-salt-induced hypertension was linked to the gut microbiota. Compared to that of the blood pressure of DOCA-salt-induced hypertensive rats without fecal transplantation, the blood pressure of normotensive rats transplanted with the fecal microbiota of DOCA-salt-induced hypertensive rats was still much lower (192.95 vs. 150.82 mmHg). These results suggested that the fecal microbiota in our hypertensive model could only partially contribute to the increased blood pressure. It has been reported that captopril treatment reshapes the gut microbiota through its sustained impacts on a unique microbial population/pattern and improves the dysregulated gut-brain axis in SHRs [43]. Our results further revealed the role of this antihypertensive drug in the alteration of the gut microbiota in the DOCA-salt hypertensive model. However, the quantitative contribution of the gut microbiota rebalancing by captopril to the reduction in blood pressure needs further elucidation, probably via FMT from rats in the CAP group to those in the DOCA group.

In previous studies related to the gut microbiota, hypertension is frequently associated with an increased F/B ratio and reduced gut microbiota diversity. Unlike those in other hypertensive models, the F/B ratio and gut microbiota diversity did not differ significantly between DOCA-salt-induced hypertensive rats and SHAM rats in this study (data not shown). The discrepancies could be due to the different types of hypertensive animal models used or an insufficient number of rats in our study. Interestingly, we first found that the phylum Proteobacteria was more abundant in the DOCA group than in the SHAM group in this model and that captopril treatment reverted its abundance relatively but not significantly. Proteobacteria is one of the most abundant phyla, comprising several known human pathogens, including *Escherichia-Shigella, Salmonella*, and *Helicobacter* [52]. A large amount of evidence currently suggests that Proteobacteria species are involved in several metabolic disorders [53–56]. *Klebsiella*, belonging to the Enterobacteriaceae family, has been reported to be abundant in hypertensive humans [28]. In our study, *Escherichia-Shigella*, a member of Enterobacteriaceae, was also enriched in the DOCA group. It is of interest that captopril administration was able to relatively rebalance its abundance in the CAP group, although the difference was not statistically significant.

The RAAS, the main pathway regulating blood pressure, is composed of three important components, renin, angiotensin, and aldosterone, in the main axis. The gut has a local RAAS that is important for the uptake of sodium and water from the colon and for the control of gut contractility [57]. Alterations in the gut microbiota might regulate blood pressure by affecting the RAAS pathway, suggesting that 20–30% of captopril is metabolized and eliminated in the gut, as reported previously [58, 59]. In addition, recent evidence indicated that sustained captopril-induced reduction in blood pressure is associated with alterations in the gut microbiota and gut pathology and permeability in the SHR [43], which further implied the effect of captopril on the gut microbiota in hypertensive rats. Our data suggested that captopril could partially restore changes in biochemical parameters, including NO and Ang II, in the RAAS and the gut microbiota in the DOCA-salt-induced hypertensive rat model.

Two abundant bacterial genera observed in the CAP group were *Bifidobacterium* and *Akkermansia*, compared to those in the SHAM and DOCA groups, in this study. *Bifidobacterium*, as a probiotic for conventional treatment of ulcerative colitis, improves the gut mucosal barrier and lowers the level of lipopolysaccharide in the intestine [60, 61]. *Akkermansia*, a mucin-degrading bacterium, can improve inflammation in diabetic and obese patients by increasing the intestinal levels of endocannabinoids [62]. The anti-inflammatory effects of captopril, demonstrated by the decreased levels of TNF-ɑ and IL-6 in DOCA-salt-induced hypertensive rats in this study, might be due to the increased abundance of *Bifidobacterium* and *Akkermansia* in the gut. *Eubacterium nodatum* and *Ruminococcus_2* were enriched in the DOCA group, but these changes were rebalanced after captopril treatment. Although the function of *Ruminococcus_2* is still unknown, *Eubacterium nodatum* has been frequently linked to chronic periodontitis [63]. It is also possible that the hypotensive effect of captopril is due to the growth inhibition of this group of bacteria in the gut. The abundance of some bacteria participating in amino acid metabolism in the gut microbiota was altered significantly between the SHAM and DOCA groups, although the differences were small. Tryptophan and glutamate metabolites are neurotransmitters directly impacting the central and peripheral nervous systems [42]. Since captopril has been indicated to alter gut-brain communication in SHRs, it is intriguing to investigate the role of captopril in the gut-brain axis in the DOCA-salt hypertensive model in the future.

In this study, we focused on the links between hypertension and the gut microbiota via the RAAS and metabolites produced in the gut flora. The drug captopril is a good tool to study their relationship and provide useful information for future work.

### Perspective

Gut dysbiosis is associated with many metabolic diseases, including hypertension. However, little is known about the role of captopril in DOCA-salt-induced hypertensive rats from the view of the gut microbiota. We present evidence in this study for the first time that (1) genera enriched in DOCA-salt-induced hypertensive rats, such as *Escherichia-Shigella, Eubacterium nodatum,* and *Ruminococcus_2*, are rebalanced in the gut microbiota by captopril treatment, (2) *Bifidobacterium* and *Akkermansia* are rebalanced in the CAP group and (3) the abundances of bacteria involved in amino acid metabolisms, such as tryptophan and arginine, are rebalanced by captopril. These observations indicate a new way to elucidate the hypotensive effects of captopril by linking the RAAS and gut microbiota.

## Supplementary information

Supplementary information
