# Supplementary material for: Hypotensive effect of captopril on deoxycorticosterone acetate-salt-induced hypertensive rat is associated with gut microbiota alteration
Source: Hypertens Res. 2021 Dec 2;45(2):270–82. doi: 10.1038/s41440-021-00796-x (PMC8766282; doi:10.1038/s41440-021-00796-x)
Supplement: Supplementary file 1 — Supplementary information [file 41440_2021_796_MOESM1_ESM.pptx]

## Slide 1
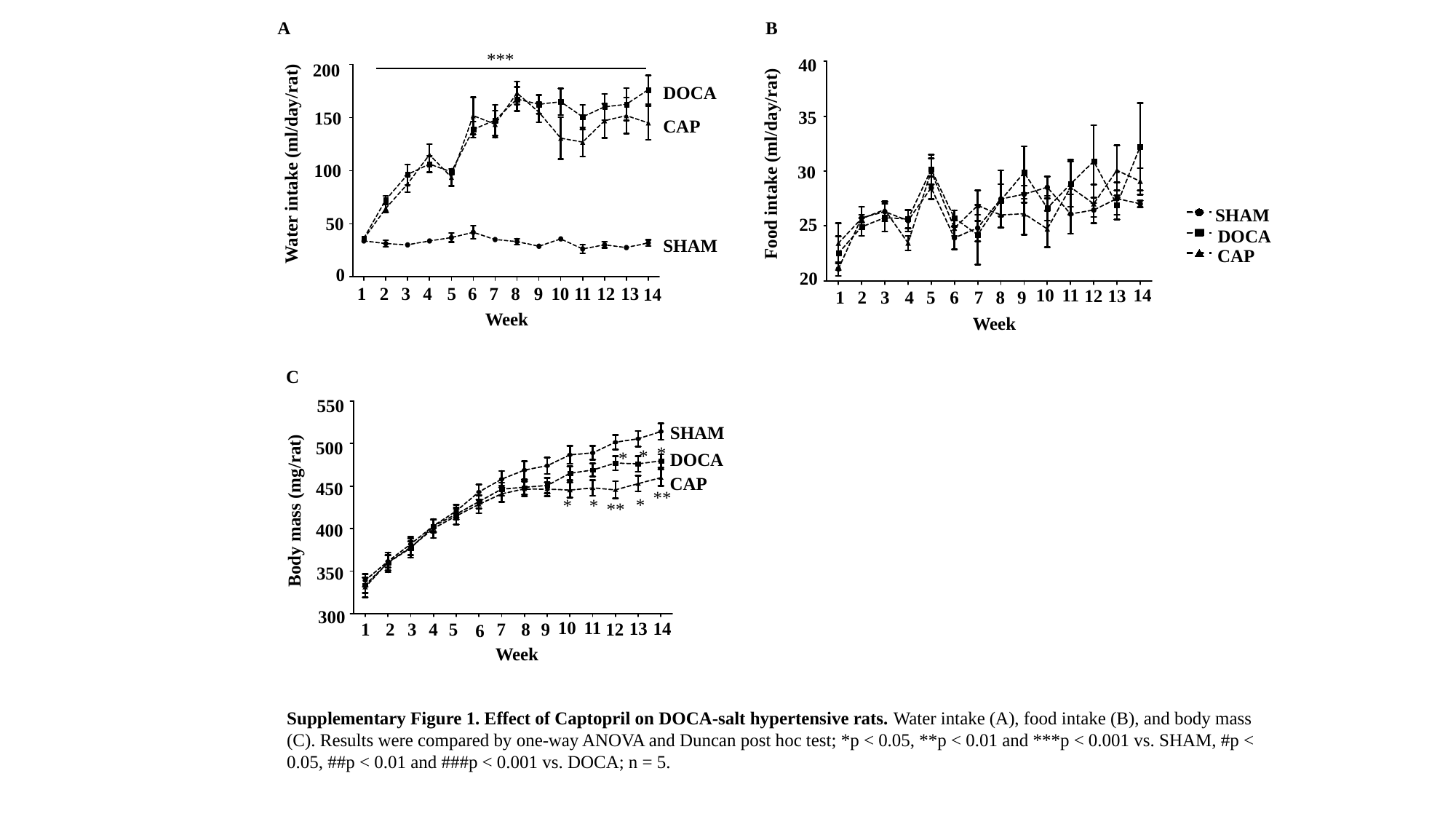

A
B
***
40
200
DOCA
35
150
CAP
Water intake (ml/day/rat)
Food intake (ml/day/rat)
100
30
SHAM
50
25
DOCA
SHAM
CAP
0
20
1
2
3
4
5
6
7
8
9
10
12
13
11
14
10
14
11
12
13
1
2
3
4
5
6
7
8
9
Week
Week
C
550
SHAM
500
*
*
*
DOCA
CAP
450
**
*
*
*
**
Body mass (mg/rat)
400
350
300
10
11
13
14
12
1
2
3
4
5
7
8
9
6
Week
Supplementary Figure 1. Effect of Captopril on DOCA-salt hypertensive rats. Water intake (A), food intake (B), and body mass (C). Results were compared by one-way ANOVA and Duncan post hoc test; *p < 0.05, **p < 0.01 and ***p < 0.001 vs. SHAM, #p < 0.05, ##p < 0.01 and ###p < 0.001 vs. DOCA; n = 5.

## Slide 2
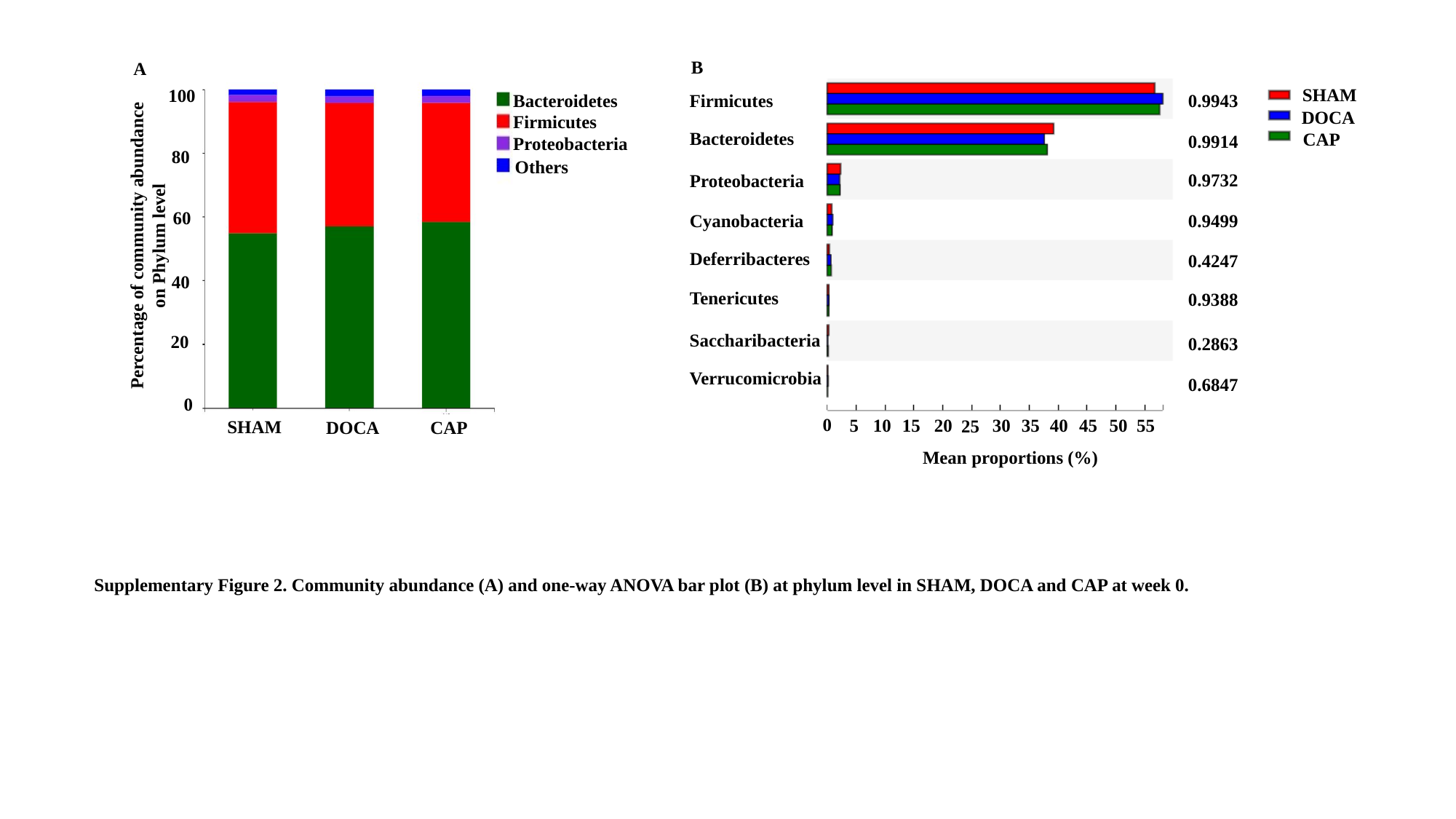

B
A
100
Bacteroidetes
Firmicutes
Proteobacteria
80
Others
60
Percentage of community abundance on Phylum level
40
20
0
SHAM
CAP
DOCA
SHAM
Firmicutes
0.9943
DOCA
Bacteroidetes
CAP
0.9914
0.9732
Proteobacteria
Cyanobacteria
0.9499
Deferribacteres
0.4247
Tenericutes
0.9388
Saccharibacteria
0.2863
Verrucomicrobia
0.6847
0
20
50
55
15
10
35
5
45
40
30
25
Mean proportions (%)
Supplementary Figure 2. Community abundance (A) and one-way ANOVA bar plot (B) at phylum level in SHAM, DOCA and CAP at week 0.

## Slide 3
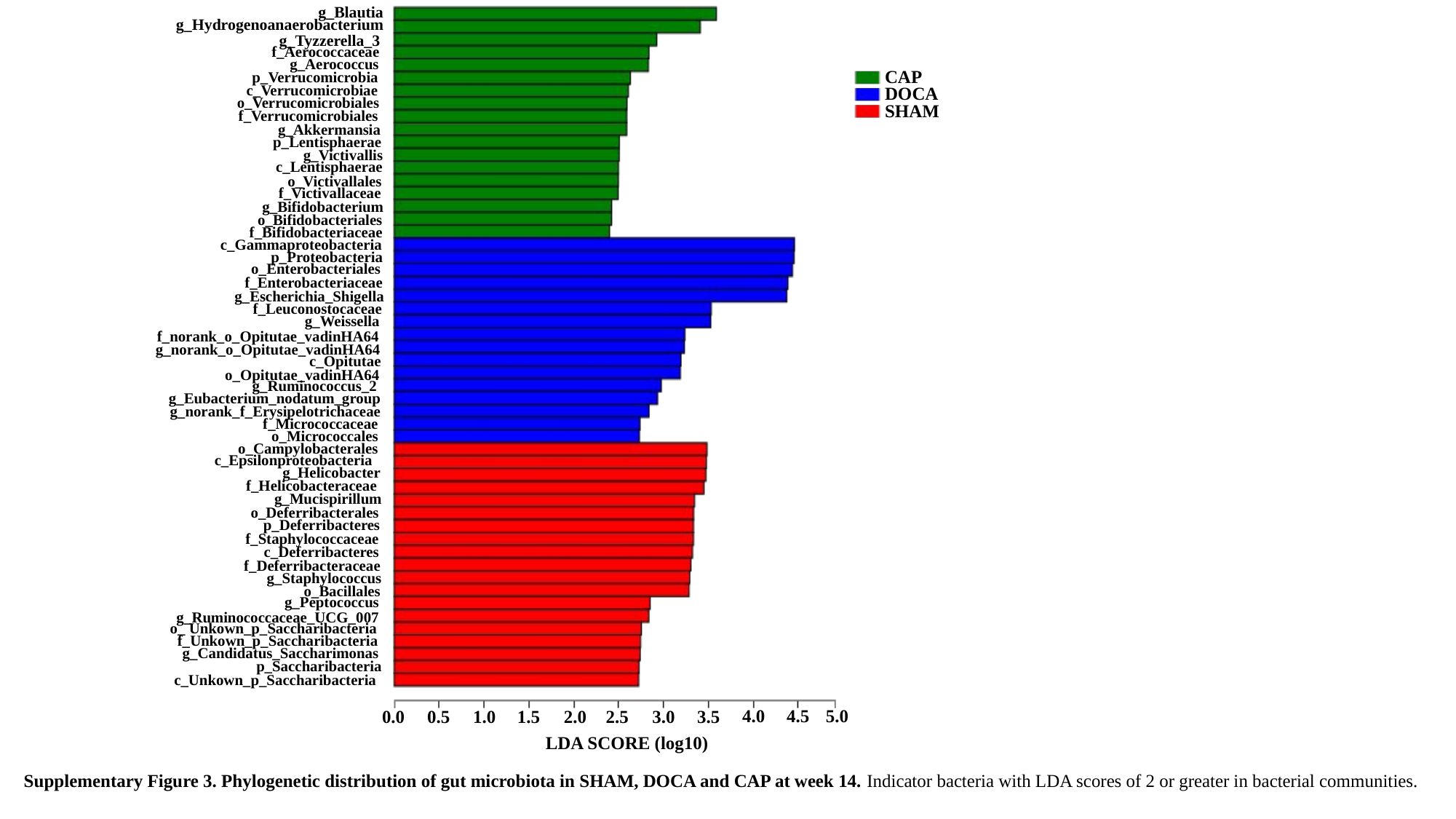

g_Blautia
g_Hydrogenoanaerobacterium
g_Tyzzerella_3
f_Aerococcaceae
g_Aerococcus
CAP
p_Verrucomicrobia
c_Verrucomicrobiae
DOCA
o_Verrucomicrobiales
SHAM
f_Verrucomicrobiales
g_Akkermansia
p_Lentisphaerae
g_Victivallis
c_Lentisphaerae
o_Victivallales
f_Victivallaceae
g_Bifidobacterium
o_Bifidobacteriales
f_Bifidobacteriaceae
c_Gammaproteobacteria
p_Proteobacteria
o_Enterobacteriales
f_Enterobacteriaceae
g_Escherichia_Shigella
f_Leuconostocaceae
g_Weissella
f_norank_o_Opitutae_vadinHA64
g_norank_o_Opitutae_vadinHA64
c_Opitutae
o_Opitutae_vadinHA64
g_Ruminococcus_2
g_Eubacterium_nodatum_group
g_norank_f_Erysipelotrichaceae
f_Micrococcaceae
o_Micrococcales
o_Campylobacterales
c_Epsilonproteobacteria
g_Helicobacter
f_Helicobacteraceae
g_Mucispirillum
o_Deferribacterales
p_Deferribacteres
f_Staphylococcaceae
c_Deferribacteres
f_Deferribacteraceae
g_Staphylococcus
o_Bacillales
g_Peptococcus
g_Ruminococcaceae_UCG_007
 o_ Unkown_p_Saccharibacteria
f_Unkown_p_Saccharibacteria
g_Candidatus_Saccharimonas
p_Saccharibacteria
c_Unkown_p_Saccharibacteria
4.0
4.5
5.0
0.5
1.0
1.5
2.0
2.5
3.0
3.5
0.0
LDA SCORE (log10)
Supplementary Figure 3. Phylogenetic distribution of gut microbiota in SHAM, DOCA and CAP at week 14. Indicator bacteria with LDA scores of 2 or greater in bacterial communities.

## Slide 4
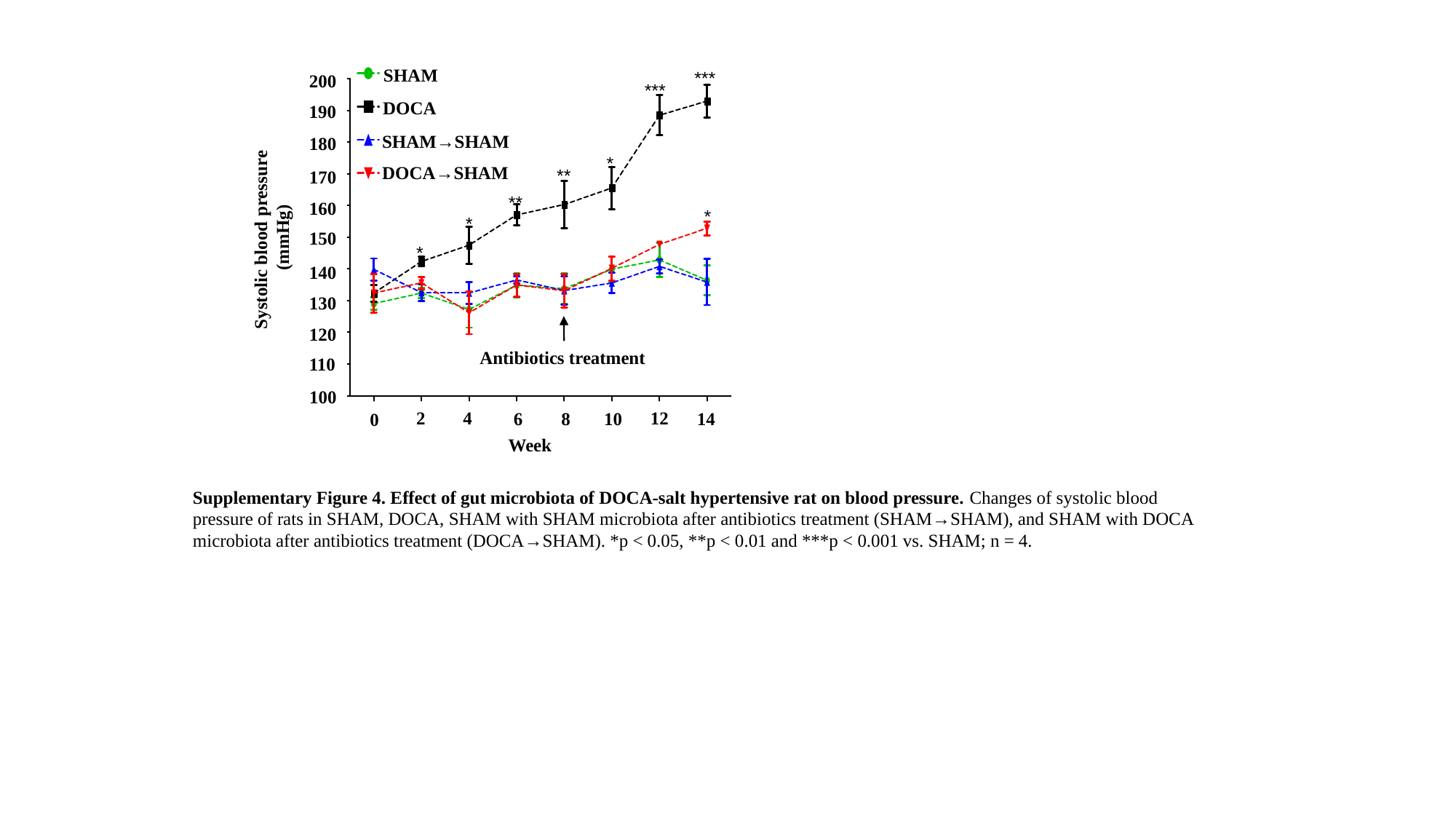

SHAM
200
DOCA
190
SHAM→SHAM
180
DOCA→SHAM
170
160
Systolic blood pressure
(mmHg)
150
140
130
120
Antibiotics treatment
110
100
2
12
4
6
8
10
14
0
Week
***
***
*
**
**
*
*
*
Supplementary Figure 4. Effect of gut microbiota of DOCA-salt hypertensive rat on blood pressure. Changes of systolic blood pressure of rats in SHAM, DOCA, SHAM with SHAM microbiota after antibiotics treatment (SHAM→SHAM), and SHAM with DOCA microbiota after antibiotics treatment (DOCA→SHAM). *p < 0.05, **p < 0.01 and ***p < 0.001 vs. SHAM; n = 4.

## Slide 5
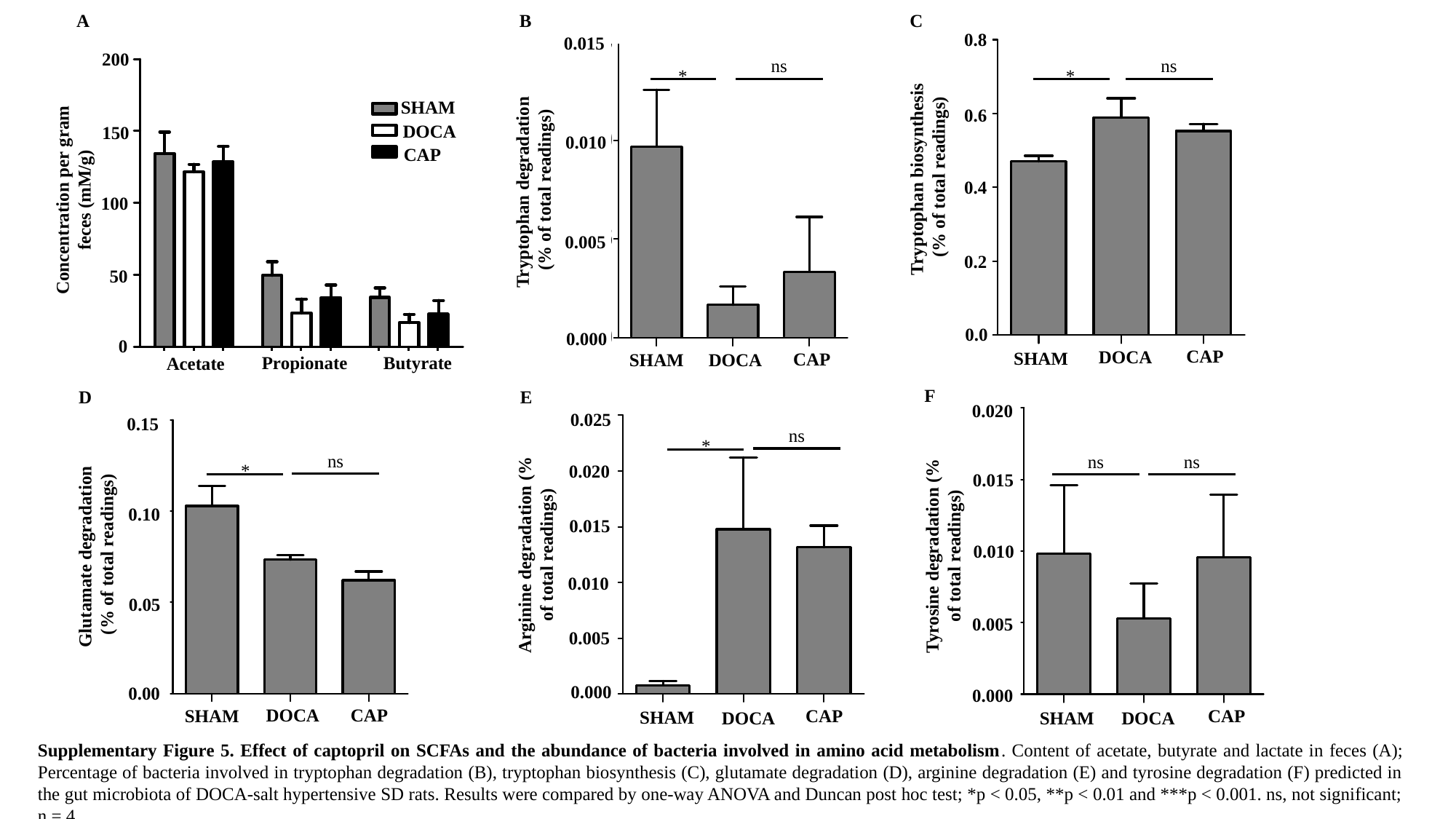

A
C
B
0.8
0.015
200
SHAM
DOCA
150
CAP
Concentration per gram feces (mM/g)
100
50
0
Propionate
Butyrate
Acetate
ns
ns
*
*
0.6
0.010
Tryptophan biosynthesis
(% of total readings)
Tryptophan degradation
(% of total readings)
0.4
0.005
0.2
0.0
0.000
CAP
DOCA
SHAM
CAP
SHAM
DOCA
F
D
E
0.020
0.025
0.15
ns
*
ns
ns
ns
*
0.020
0.015
0.10
0.015
Glutamate degradation
(% of total readings)
Arginine degradation (% of total readings)
Tyrosine degradation (% of total readings)
0.010
0.010
0.05
0.005
0.005
0.000
0.00
0.000
DOCA
CAP
SHAM
CAP
CAP
SHAM
DOCA
DOCA
SHAM
Supplementary Figure 5. Effect of captopril on SCFAs and the abundance of bacteria involved in amino acid metabolism. Content of acetate, butyrate and lactate in feces (A); Percentage of bacteria involved in tryptophan degradation (B), tryptophan biosynthesis (C), glutamate degradation (D), arginine degradation (E) and tyrosine degradation (F) predicted in the gut microbiota of DOCA-salt hypertensive SD rats. Results were compared by one-way ANOVA and Duncan post hoc test; *p < 0.05, **p < 0.01 and ***p < 0.001. ns, not significant; n = 4.
